# Association between Myopia, Biometry and Occludable Angle: The Jiangning Eye Study

**DOI:** 10.1371/journal.pone.0165281

**Published:** 2016-10-20

**Authors:** Xiaohong Liu, Hehua Ye, Qi Zhang, Xuan Cai, Wenjing Yu, Siyi Yu, Tianyu Wang, Wuyi Lu, Xiang Li, Peiquan Zhao

**Affiliations:** 1 Department of Ophthalmology, Xinhua Hospital, Shanghai Jiao Tong University School of Medicine, Shanghai, P. R. China; 2 Department of Ophthalmology, Shanghai Jiao Tong University Affiliated Sixth People's Hospital, Shanghai, P. R. China; Bascom Palmer Eye Institute, UNITED STATES

## Abstract

**Background:**

Myopia subjects usually have long axial lengths and are predisposed to deep anterior chamber depths and open angles. Jiangning eye study is a randomized, population based study in Shanghai to determine whether myopia has an effect on the prevalence of occludable angles.

**Methods:**

A total of 2478 residents aged 50 years and over were randomly selected, and 2044 (82.5%) individuals of them participated in ophthalmic examinations between November 2012 and February 2013. Eye examinations included autorefraction, noncontact tonometry, measurement of axial length, anterior chamber depth, lens thickness and spectral domain optical coherence tomography.

**Results:**

The crude prevalences of iris trabecular meshwork contact in Jiangning Chinese individuals with myopia, emmetropia, and hyperopia are 1.25%, 6.44%, and 7.43%, respectively. There is also iris trabecular meshwork contact and shallow anterior chamber depth in myopia subjects even with long axial lengths.

**Conclusions:**

The prevalence of iris trabecular meshwork contact in myopia subjects is lower than their emmetropia or hyperopia counterparts. Axial length is an important factor associate with occludable angle.

## Introduction

Glaucoma is the second leading cause of irreversible blindness, affecting more than 60 million patients worldwide. Approximately half of the patients with primary angle closure glaucoma (PACG) live in China, and angle closure glaucoma usually results in more severe vision loss [1]. It is therefore important to assess the prevalence of occludable angles and the likely risk factors for angle closure glaucoma in Chinese people.

It is well known that angle closure glaucoma is correlated with many risk factors, such as hyperopia [2], shallow anterior chamber depth (ACD) [3], short axial length (AL) [4], increased lens thickness (LT), and female gender [5]. Most of these risk factors are associated with shallow anterior chamber depth to some extent [6]. For example, hyperopia is usually associated with the presence of shallow ACD and short axial length, and ACD decreases with increasing lens thickness. Therefore, shallow anterior chamber depth is considered the most important risk factor for angle closure glaucoma [6].

Myopia subjects usually have long axial lengths and are predisposed to deep ACDs and open angles. However, some recent studies have reported the presence of angle closure glaucoma in myopia subjects. Kai-Ling Yong reported that almost one quarter of angle closure patients were myopic. Yong also reported that although myopic angle-closure subjects had longer axial lengths and vitreous lengths, there were no significant differences in ACD, lens thickness or lens vault between these patients and their emmetropic and hyperopic counterparts [7].

It is well known that Chinese individuals have a high prevalence of both myopia and angle closure [8–10]. If it is true that subjects with myopia have a low chance of developing angle closure, the prevalence of occludable angles in Chinese people may decrease with increasing myopia prevalence. However, He Ming Guang reported that the increasing prevalence of myopia has a minimal impact on the prevalence of occludable angles [11]. However, the aforementioned study was not a randomized study, and the prevalence of myopia was altered by the selection of a certain proportion of myopia subjects, which inevitably results in selection bias.

Some limitations of the Yong and He’s study were that it was not randomized or based on the general population. In addition, the study populations comprised Asian or Guangzhou individuals, and there are no other studies about the association of myopia and occludable angles in China. Because China’s area is large, some internal epidemiological differences in its population may be greater than the corresponding differences among countries in Europe. Therefore, we designed a randomized, population based study in Shanghai to determine whether myopia has an effect on the prevalence of occludable angles. In our study, an occludable quadrant was defined as iris trabeculary meshwork contact (ITC) in a picture acquired by spectral domain optical coherence tomography (SD-OCT) [12]. SD-OCT is a noncontact instrument and with a good sensitivity in detecting occludable angle. Nolan WP reported that in dark conditions, AS-OCT identified 98% of those subjects found to have angle closure by gonioscopy [13].

## Materials and Methods

### Study population

The Jiangning eye study is a cross-sectional, population-based study of urban Chinese individuals aged 50 years and older in the Jiangning sub-district of Shanghai in the Southeast of China. After excluding vacant households, 2478 residents were randomly selected using a stratified, clustered, and multistage sampling technique, with probabilities proportionate to the size of the population of each cluster. Of the 2478 randomly selected individuals, 2044 (82.5%) people participated in an ophthalmic examination between November 2012 and February 2013. The main reasons for non-participation at examination stage were below: too old, bad health, not at home and busy, etc. The study was approved by the Ethics Committee of Xinhua Hospital, Shanghai Jiao Tong University School of Medicine (approval Number: XHEC-C-2012-014), and the study was conducted in accordance with the tenets of the Declaration of Helsinki. Written informed consent was obtained from all participants before enrolment in the study. The details of the study are described in Ye Hehua’s paper [14]. The data is stored in Xinhua hospital and the author have no access to information that could identify individual participants during or after data collection.

### Study procedures

Eye examinations were conducted according to a standardized protocol that included visual acuity measurements with ETDRS (early treatment diabetic retinopathy study) charts and recorded in each eye separately with best corrected acuity, autorefraction (KR8900; Topcon, Tokyo, Japan), noncontact tonometry (CT80A; Topcon), slit lamp biomicroscopy (SL1E; Topcon), direct ophthalmoscopy (YE6F; 66Vision, Suzhou, China), measurement of axial length, anterior chamber depth, lens thickness and corneal thickness (A-Scan model SW1000, Suoer, Tianjin, China); and spectral domain optical coherence tomography (OCT) (Topcon 3DOCT 2000; Topcon). Digital images of angle photographs were analysed with the 3D OCT2000 integrated software package (Topcon). Every instrument was operated by the same operator.

#### SD-OCT

All subjects underwent anterior segment OCT. Four scans of the angle of each eye were obtained (at the 12-, 6-, 3-, and 9-o’clock positions) with the SD-OCT in the dark and operated by the same skilled technician. The subject’s fixation was directed by the fixation light beside the instrument [15].

#### A-Scan biometry

If the standard deviation (SD) of these measurements was 0.12 mm or greater, all six readings were discarded, and the process was repeated until the SD was less than 0.12 mm.

#### SD-OCT picture analysis

Cases with previous surgery that changed the anterior segment structure (e.g., cataract or glaucoma surgery) were excluded [16]. We defined a closed quadrant of the anterior chamber angle as the presence of any contact between the iris and the angle wall anterior to the scleral spur, which we termed iris trabecular meshwork contact (ITC) [17]. And we also defined that Level of ITC-1 indicates that there is only one quadrant with ITC in one eye; ITC-2 indicates that there are at least 2 quadrants with ITC in one eye; ITC-sum indicates that there is at least one quadrant with ITC in one eye (ITC-sum = ITC-1 + ITC-2).

### Statistical analysis

We conducted our analyses on the left eye. A chi-square test was used to compare demographic characteristics between subjects with different refraction sphere grades. Logistic regression analysis was performed to determine the risk factors for an occludable angle using odds ratio (OR) estimates. For multivariate logistic regression analysis, we included risk factors with *P*<.05 in the multivariate adjusted model. Student’s *t* test was used to compare the biometric characteristics between different refraction sphere grades. Multivariate linear regression was used to analyse the association between anterior chamber depth and biometry parameters. Statistical analyses were performed using a commercially available statistical software package, SPSS12.0. All *P* values were two sided and were considered statistically significant when less than 0.05.

## Results

### Prevalences of ITC at different refraction sphere grades

After excluding subjects who had surgery that altered the anterior segment structure, 1859 eyes remained for analysis. When the data of the left eye of some subjects was missing, we chose the right eye instead. So there were 1801 left eyes and 58 right eyes remained for analysis. It is shown in Table 1 that mean age and sex ratio of myopia, emmetropia, and hyperopia groups were similar, which decrease the bias due to age and sex. Table 1 also shows that the crude prevalences of ITC-sum in the myopia, emmetropia, and hyperopia groups were 1.25%, 6.44%, and 7.43%, respectively. And the crude prevalences of ITC-2 in the myopia, emmetropia, and hyperopia group were 0.54%, 3.79%, and 3.09%, respectively. In subjects with ITC-2, the numbers of high, moderate, and low myopia subjects were 0, 1, and 2, respectively, which means that there were no high myopia subjects with ITC in at least 2 quadrants of the chosen eye. There were significant differences in ITC prevalence among the myopia group and the emmetropia and hyperopia groups. There was no significant difference in ITC prevalence between the emmetropia and hyperopia groups.

**Table 1:** Prevalence of iris trabecular meshwork contact (ITC) in the myopia, emmetropia, and hyperopia groups.

Refraction sphere	*n*	Mean age (SD)	Male (%)	No ITC	ITC-sum	
					ITC-1	ITC-2
				*n* (%)	*n* (%)	*n* (%)
Myopia*						
High myopia (diopter ≥ -6.0D)	170	62.27 (7.68)	40.00	169 (99.41)	1 (0.59)	0
Moderate myopia (-6.0D < diopter ≤ -3.0D)	141	62.95 (8.13)	45.39	138 (97.87)	2 (1.42)	1 (0.71)
Low myopia (-3.0D < diopter ≤ -0.5D)	247	62.83 (9.13)	43.72	244 (98.79)	1 (0.40)	2 (0.81)
Total	558	62.69 (8.45)	43.01	551 (98.75)	4 (0.72)	3 (0.54)
**Emmetropia** (-0.5D < diopter < 0.5D)	264	62.27 (9.35)	39.39	247 (93.56)	7 (2.65)	10 (3.79)
**Hyperopia**						
Low hyperopia (0.5D ≤ diopter < 3.0D)	848	63.93 (9.49)	44.69	788 (92.92)	36 (4.25)	24 (2.83)
Moderate hyperopia (3.0D ≤ diopter < 6.0D)	165	72.56 (9.38)	47.27	150 (90.91)	8 (4.85)	7 (4.24)
High hyperopia (diopter ≥ 6.0D)	24	75.04 (11.67)	50.00	22 (91.67)	1 (4.17)	1 (4.17)
Total	1037	65.56 (10.13)	45.23	960 (92.57)	45 (4.34)	32 (3.09)

Level of significance set at *p*<0.05; statistical significance tested with chi square test.

* There were significant differences in ITC prevalence among the myopia group and the emmetropia and hyperopia groups.

ITC-1 indicates that there is only one quadrant with ITC in the left eye; ITC-2 indicates that there are at least 2 quadrants with ITC in the left eye. ITC-sum indicates that there is at least one quadrant with ITC in the left eye (ITC-sum = ITC-1 + ITC-2).

There is no missing data in Table 1.

### Risk factors for iris trabecular meshwork contact

Table 2 shows that age, sex, refraction sphere, axial length, lens thickness, and anterior chamber depth are risk factors of ITC and keratometry, height, weight, waistline, IOP are not risk factors of ITC when age and sex adjusted. However, after multivariate adjustment, age, axial length, lens thickness and anterior chamber depth are also risk factors of ITC but sex, refraction sphere are not independent risk factors of ITC.

**Table 2:** Age and sex or multivariate adjusted odds ratios of risk factors for the development of iris trabecular meshwork contact (ITC).

	*n* (missing)	Age and sex adjusted			Multivariate adjusted**		
		OR	95% CI	P-value	OR	95% CI	P-value
**Age, y**							
50–59	729 (0)	0.38	(0.23, 0.62)	0.00*	0.48	(0.28, 0.82)	0.01*
60–69	638 (0)	0.59	(0.37, 0.95)	0.03*	0.84	(0.51, 1.39)	0.50
≥ 70	492 (0)	1 (reference)			1 (reference)		
**Sex**							
Male	813 (0)	0.50	(0.32, 0.78)	0.00*	0.64	(0.40, 1.02)	0.06
Female	1046 (0)	1 (reference)			1 (reference)		
**Refraction sphere (diopter)**	1859 (0)	1.19	(1.10, 1.29)	0.00*	0.97	(0.87, 1.08)	0.56
**Keratometry**	1859 (4)	1.03	(0.90, 1.18)	0.65			
**Height (cm)**	1859 (3)	0.98	(0.94, 1.01)	0.14			
**Weight (kg)**	1859 (3)	1.01	(0.98, 1.03)	0.68			
**Waistline (cm)**	1859 (3)	1.00	(0.98, 1.02)	0.77			
**IOP (mmHg)**	1859 (21)	1.05	(0.98, 1.12)	0.14			
**Axial length (mm)**	1859 (0)	0.41	(0.33, 0.52)	0.00*	0.46	(0.35, 0.61)	0.00*
**Lens thickness (mm)**	1859 (0)	3.27	(1.78, 5.60)	0.00*	1.93	(1.01, 3.68)	< 0.05*
**ACD (mm)**							
< 2.80	685 (0)	5.08	(2.86, 9.03)	0.00*	1.99	(1.07, 3.73)	0.03*
2.80–2.99	410 (0)	1.67	(0.80, 3.50)	0.18	1.07	(0.50, 2.28)	0.87
≥ 3.00	764 (0)	1 (reference)			1 (reference)		

Level of significance set at *p*<0.05; statistical significance tested with logistic regression analysis.

*p<0.05,

**Multivariate adjustment was performed for age, sex, refraction sphere, axial length, lens thickness, and anterior chamber depth.

ACD = central anterior chamber depth, IOP = intraocular pressure.

### Biometry and refraction sphere

Table 3 shows that there were significant differences in axial length and anterior chamber depth between any two of the myopia, emmetropia, and hyperopia groups. There was no significant difference in lens thickness between the myopia and the emmetropia groups, but there was significant difference in lens thickness (LT) between the hyperopia and the myopia and emmetropia groups. We also found that the axial length and anterior chamber depth of the myopia subjects were greater than those of the emmetropia or hyperopia subjects. In addition, ACD and AL also increased when myopia increased from low to high.

**Table 3:** Biometry parameters at different refraction sphere grades.

Refraction sphere	*n*	Mean age (SD)	Male (%)	ACD	LT	AL
Myopia*						
High myopia (diopter ≥ -6.0D)	170	62.27 (7.68)	40.00	3.14 (0.35)	4.59(0.34)	27.43(1.76)
Moderate myopia (-6.0D < diopter ≤ -3.0D)	141	62.95 (8.13)	45.39	3.10 (0.34)	4.55 (0.33)	25.42 (1.16)
Low myopia (-3.0D < diopter ≤ -0.5D)	247	62.83 (9.13)	43.72	3.03 (0.33)	4.62 (0.36)	24.23 (1.02)
Total	558	62.69 (8.45)	43.01	3.08 (0.34)	4.59 (0.35)	25.50 (1.89)
**Emmetropia** (-0.5D < diopter < 0.5D)	264	62.27 (9.35)	39.39	2.93 (0.31)	4.60 (0.32)	23.47 (1.01)
**Hyperopia**						
Low hyperopia (0.5D ≤ diopter < 3.0D)	848	63.93 (9.49)	44.69	2.84 (0.33)	4.70 (0.35)	23.21 (1.02)
Moderate hyperopia (3.0D ≤ diopter < 6.0D)	165	72.56 (9.38)	47.27	2.80 (0.33)	4.77 (0.38)	22.87 (0.96)
High hyperopia (diopter ≥ 6.0D)	24	75.04 (11.67)	50.00	2.83 (0.31)	4.70 (0.39)	22.70 (1.75)
Total	1037	65.56 (10.13)	45.23	2.83 (0.33)	4.71 (0.35)	23.14 (1.04)

Level of significance set at *p*<0.05; statistical significance tested with student’s *t* test between myopia, emmetropia and hyperopia groups. ACD = central anterior chamber depth, AL = axial length, LT = lens thick.

There is no missing data in Table 3.

### Association between ACD and biometry

Table 4 shows that in multiple liner regression analyses, ACD = 1.937 + 0.107AL − 0.335LT + 0.013 (refraction sphere). Anterior chamber depth increased noticeably with increasing axial length and decreased with increasing lens thickness. The effect of the refraction sphere on ACD was so small that it could be ignored in most cases.

**Table 4:** Association between anterior chamber depth and axial length, lens thickness and refraction sphere.

Independent variable	coefficient	*t*	*p*
**Axis length (mm)**	0.107	17.429	0.000*
**Lens thickness (mm)**	-0.335	-17.450	0.000*
**Refraction sphere (diopter)**	0.013	4.964	0.000*

Dependent variable: central anterior chamber depth. Level of significance set at *p*<0.05; statistical significance tested with multivariate linear regression analysis;

*p<0.05.

### Characteristics of myopic subjects with ITC

It is shown in Table 5 that there were 7 subjects with myopia (high myopia: 1; moderate myopia: 3; low myopia: 3) who had at least one quadrant of ITC and all of these 7 subjects had shallow anterior chamber depths. In six of these 7 subjects, the ACDs were not greater than 2.60 mm, and only one subject had a ACD greater than 2.80 mm. It is also shown that subject 1 and 4 had long axial lengths greater than 26.00 mm, subject 2 and 7 had moderate axial lengths between 23.00 and 24.00 mm, and subject 3, 5, 6 have short axial lengths less than23.00 mm. In the subjects with long axial length in Table 5 (subject 1, 4), the subjects usually had thick lens (LT = 5.07, 4.76 mm). In the subjects with moderate axial lengths (subject 2 and 7) in Table 5, subject 2 have thick lens too (LT = 5.12m). In the subjects with short axial length in Table 5 (subject 3, 5, 6), subject 3 have axial length less than 22.00mm, subject 5 have thick lens (LT = 4.97); and subjects 6 have lens shifted forward (RLP = 0.177).

**Table 5:** Summary of iris trabecular meshwork contact (ITC) subjects with myopia.

Subjects No.	Age	Sex	ITC-sum	Diopter	ACD	AL	LT	RLP
**1**	68	F	1	-6.75	2.38	26.05	5.07	0.189
**2**	83	F	4	-4.13	2.34	23.54	5.12	0.208
**3**	79	F	1	-5.00	2.60	21.58	4.41	0.223
**4**	60	M	1	-3.50	2.55	26.20	4.76	0.188
**5**	84	M	3	-1.75	2.24	22.47	4.97	0.210
**6**	77	M	2	-1.38	2.24	22.96	3.64	0.177
**7**	53	M	1	-0.50	2.82	23.79	4.35	0.211

ITC-sum indicates there is at least one quadrant of iris trabecular meshwork contact, ACD = central anterior chamber depth, AL = axial length, LT = lens thick, RLP = relative lens position, RLP = (ACD+0.5LT)/AL.

## Discussion

### Prevalences of ITC at different refraction sphere grades and risk factors

In our study, there were 45 subjects who had at least 2 quadrants with ITC in the chosen eye. In these 45 subjects, 3 subjects had myopia, 10 subjects had emmetropia, and 32 subjects had hyperopia. In subjects with an occludable angle (ITC = 2), approximately 6.7%, 22.2%, and 71.1% of the subjects had myopia, emmetropia, and hyperopia, respectively. In subjects with occludable angle (ITC = 2) in our study, the number of subjects with hyperopia was more than 10 times the number of subjects with myopia, which means that myopia accounts for only a small part of subjects with an occludable angle. Our result is consistent with studies by Chakravarti T and Barkana Y [18, 19]. Chakravarti reported 6 cases of myopia in 322 cases of primary angle closure [18], and Barkana reported 20 subjects with angle closure in a database of 17,938 persons with high myopia [19]. However, Kai-lin Yong reported that almost one quarter of angle-closure patients were myopic [7]. He Ming Guang reported that the increasing prevalence of myopia has minimal impact on the prevalence of occludable angles, with myopia increasing from 10% to 60% and narrow angle prevalence decreasing from 11.1% to 9.6% [11]. The difference between our study and Kai-lin Yong’s study may associate with subject selection bias. Kai-lin Yong’s study was neither a population-based nor a randomized study, whereas our study is randomized and population-based.

In addition, there are three issues that may correlate with the difference between our study and the He Ming Guang’s study. First, He Ming Guang’s study was not randomized, and the proportion of myopia subjects in the group was designed by the author, which will result in selection bias inevitably. Second, the prevalence of occludable angles may be different between these two areas of China. The prevalence of occludable angles in Guangzhou may be higher than that in Shanghai. For example, shallow ACD is the most important risk factor for occludable angle, and the ACD in He Ming Guang’s study was shallower than that in our study. The mean ACD in He Ming Guang’s study increased from 2.68 mm to 2.74 mm when the prevalence of myopia increased from 10% to 60% [11], whereas in our study, the mean ACD increased from 2.83 (hyperopia group) to 3.08 (myopia group). This shows that the upper limit of the mean ACD in their study (2.74 mm, 60%myopia) was still shallower than the lower limit of the mean ACD (2.83 mm, 100% hyperopia) in our study. Third, the different detection methods (gonoiscopy and AS-OCT) of the two studies may also contribute to the difference. Some studies have reported that SD-OCT detects fewer closed angle cases than gonoiscopy [15]. Whether the difference between the two studies is based on equipment differences or geographic differences worth further study.

Table 1 shows that the prevalence of occludable angles in myopia subjects is lower than in emmetropia and hyperopia subjects. Because age is an important risk factor, we calculated the age adjusted prevalences to avoid the influence of age. After age-adjusted, the prevalences of ITC-sum in the myopia, emmetropia, and hyperopia group were 1.25%, 6.91%, and 7.19%, respectively and the prevalences of ITC-2 in the myopia, emmetropia, and hyperopia group were 0.54%, 4.05%, 2.93%, respectively. The age composition of the myopia group was the standard for age adjustment. This means that even after age adjustment, the prevalence of occludable angles in myopia subjects is lower than in emmetropia and hyperopia subjects. All in all, myopia may be a protective factor for occludable angles.

The multivariate logic regression analysis also showed a linear relationship between the refraction sphere and occludable angles when adjusting for age and sex. However, after axial length was adjusted, linear regression between the refraction sphere and occludable angles was no longer apparent. In other words, axial length, rather than refraction sphere, correlated with an occludable angle [4, 20]. This may be associated with there is collinearity between refraction sphere and axial length [10].

### Association between ACD and biometry

Our study also shows a linear relationship among ACD and axial length and lens thickness. In our study, ACD increases when axial length increases and decreases with increasing lens thickness, which has also been reported by others [21, 22]. Although the regression coefficient of lens thickness is 3 times the regression coefficient of AL, AL may be the most important factor in Table 4 that effect the ACD in most cases. Because the lens thickness didn’t change much in most cases. Today, cataract surgery is performed earlier than in the past; therefore, it is rare to see too thick lens. However, the high prevalence of myopia resulting in increased AL is more common and often great, so AL is the most important factor that effects the ACD in most cases. As we all know that apart from some cases with refractive or index myopia, most moderate and high myopia cases are characterized by increased axial lengths.

### Characteristics of myopic subjects with ITC

From Table 5 we can find that long, moderate and short axial lengths were all present in myopia subjects with ITC, this means that myopia subjects don’t necessarily have long axial lengths. In some index myopia subjects, the axial length may be not long or short. We can also learn from subject 1 and 4 in Table 5 that when the effect of lens thickening is greater than the axial length increasing, the ACD becomes shallow, and the angle becomes occludable, even with an increased axial length. However, this situation is rare and occurs only when the lens becomes very thick, so the prevalence of occludable angles is lower in myopia subjects than in hyperopia subjects. It is also shown in subject 6 that the ACD of myopia subjects is shallow too when the lens is not thick but shifted forward (RLP = 0.177). To sum up, the data in our study shows that in myopia subjects, short AL, thick lens and shifted forward lens are main risk factors for angle closure.

In summary, we conclude that the crude prevalences of iris trabecular meshwork contact (ITC-sum) in Jiangning Chinese individuals with myopia, emmetropia, and hyperopia are 1.25%, 6.44%, and 7.43%, respectively. Subjects with myopia have a lower ITC prevalence than subjects with emmetropia or hyperopia. Subjects with myopia usually have a deep anterior chamber depth, and anterior chamber depth increase with increasing axial length if the lens thickness is not great. However, ITC still occurs in myopia subjects with long axial lengths. Axial myopia subjects with ITC usually have shallow anterior chamber depths and thick lenses or a relative forward shift of the lens.

## Supporting Information

S1 Dataset The original data saved in SPSS format. (SAV)

## References

[1] Quigley HA, Broman AT. The number of people with glaucoma worldwide in 2010 and 2020. Br J Ophthalmol. 2006;90:262–267. 10.1136/bjo.2005.081224 16488940 PMC1856963

[2] Dandona L, Dandona R, Mandal P, Srinivas M, John RK, McCarty CA, et al Angle-closure glaucoma in an urban population in southern India. The Andhra Pradesh eye disease study. Ophthalmology. 2000;107:1710–1716. 10964834 10.1016/s0161-6420(00)00274-8

[3] Bonomi L, Marchini G, Marraffa M, Bernardi P, De Franco I, Perfetti S, et al Epidemiology of angle-closure glaucoma: prevalence, clinical types, and association with peripheral anterior chamber depth in the Egna-Neumarket Glaucoma Study. Ophthalmology. 2000;107:998–1003. 10811096 10.1016/s0161-6420(00)00022-1

[4] Thapa SS, Paudyal I, Khanal S, Paudel N, van Rens GH. Comparison of axial lengths in occludable angle and angle-closure glaucoma—the Bhaktapur Glaucoma Study. Optom Vis Sci. 2011;88:150–154. 10.1097/OPX.0b013e318205e320 21150676

[5] Lavanya R, Wong TY, Friedman DS, Aung HT, Alfred T, Gao H, et al Determinants of angle closure in older Singaporeans. Arch Ophthalmol (Chicago, Ill: 1960). 2008;126:686–691. 10.1001/archopht.126.5.686 18474780

[6] Xu L, Cao WF, Wang YX, Chen CX, Jonas JB. Anterior chamber depth and chamber angle and their associations with ocular and general parameters: the Beijing Eye Study. Am J Ophthalmol. 2008;145(5)929–936. 10.1016/j.ajo.2008.01.004 18336789

[7] Yong KL, Gong T, Nongpiur ME, How AC, Lee HK, Cheng L, et al Myopia in asian subjects with primary angle closure: implications for glaucoma trends in East Asia. Ophthalmology. 2014;121:1566–1571. 10.1016/j.ophtha.2014.02.006 24679835

[8] Morgan IG, Ohno-Matsui K, Saw SM. Myopia. Lancet (London, England). 2012;379:1739–1948. 10.1016/S0140-6736(12)60272-4 22559900

[9] Liang YB, Lin Z, Vasudevan B, Jhanji V, Young A, Gao TY, et al Generational difference of refractive error in the baseline study of the Beijing Myopia Progression Study. Br J Ophthalmol. 2013;97:765–769. 10.1136/bjophthalmol-2012-302468 23590854 PMC3664384

[10] He M, Huang W, Li Y, Zheng Y, Yin Q, Foster PJ. Refractive error and biometry in older Chinese adults: the Liwan eye study. Invest Ophthalmol Vis Sci. 2009;50:5130–5136. 10.1167/iovs.09-3455 19553618

[11] Jin G, Ding X, Guo X, Chang BH, Odouard C, He M. Does Myopia Affect Angle Closure Prevalence. Invest Ophthalmol Vis Sci. 2015;56:5714–5719. 10.1167/iovs.15-16914 26313306

[12] Nolan WP, See JL, Chew PT, Friedman DS, Smith SD, Radhakrishnan S, et al Detection of primary angle closure using anterior segment optical coherence tomography in Asian eyes. Ophthalmology. 2007;114:33–39. 10.1016/j.ophtha.2006.05.073 17070597

[13] Nolan WP, See JL, Chew PT, Friedman DS, Smith SD, Radhakrishnan S, et al Detection of primary angle closure using anterior segment optical coherence tomography in Asian eyes.Ophthalmology. 2007;114:33–39. 10.1016/j.ophtha.2006.05.073 17070597

[14] Ye H, Zhang Q, Liu X, Cai X, Yu W, Yu S, et al Prevalence of age-related macular degeneration in an elderly urban chinese population in China: the Jiangning Eye Study. Invest Ophthalmol Vis Sci. 2014;55:6374–6380. 10.1167/iovs.14-14899 25190650

[15] Quek DT, Narayanaswamy AK, Tun TA, Htoon HM, Baskaran M, Perera SA, et al Comparison of two spectral domain optical coherence tomography devices for angle-closure assessment. Invest Ophthalmol Vis Sci. 2012;53:5131–5136. 10.1167/iovs.12-10132 22786910

[16] He M, Foster PJ, Ge J, Huang W, Wang D, Friedman DS, et al Gonioscopy in adult Chinese: the Liwan Eye Study. Invest Ophthalmol Vis Sci. 2006;47:4772–4779. 10.1167/iovs.06-0309 17065487

[17] Sakata LM, Lavanya R, Friedman DS, Aung HT, Gao H, Kumar RS, et al Comparison of gonioscopy and anterior segment ocular coherence tomography in detecting angle closure in different quadrants of the anterior chamber angle. Ophthalmology. 2008;115:769–774. 10.1016/j.ophtha.2007.06.030 17916377

[18] Chakravarti T, Spaeth GL. The prevalence of myopia in eyes with angle closure. J Glaucoma. 2007;16:642–643. 10.1097/IJG.0b013e318064c803 18091187

[19] Barkana Y, Shihadeh W, Oliveira C, Tello C, Liebmann JM, Ritch R. Angle closure in highly myopic eyes. Ophthalmology. 2006;113:247–254. 10.1016/j.ophtha.2005.10.006 16427698

[20] George R, Paul PG, Baskaran M, Ramesh SV, Raju P, Arvind H, et al Ocular biometry in occludable angles and angle closure glaucoma: a population based survey. Br J Ophthalmol. 2003;87:399–402. 12642298 10.1136/bjo.87.4.399 PMC1771625

[21] Hsieh YT. Collinearity in multivariate analysis for anterior chamber depth and chamber angle with other parameters. Am J Ophthalmol. 2009;147:1108; author reply -9. 10.1016/j.ajo.2009.03.007 19463551

[22] Park SH, Park KH, Kim JM, Choi CY. Relation between axial length and ocular parameters. Ophthalmologica. 2010;224:188–193. 10.1159/000252982 19864929

